# The development and validation of a multi-dimensional medical students’ learning self-efficacy questionnaire for clinical education

**DOI:** 10.1080/10872981.2025.2534053

**Published:** 2025-07-20

**Authors:** Chia-Ter Chao, Jyh-Chong Liang

**Affiliations:** a Division of Nephrology, Department of Internal Medicine, National Taiwan University Hospital and National Taiwan University College of Medicine, Taipei, Taiwan; b Division of Nephrology, Department of Internal Medicine, Min Sheng General Hospital, Taoyuan, Taiwan; c Graduate Institute of Medical Education and Bioethics, National Taiwan University College of Medicine, Taipei, Taiwan; d Graduate Institute of Toxicology, National Taiwan University College of Medicine, Taipei, Taiwan; e Program of Learning Sciences and Institute for Research Excellence in Learning Sciences, National Taiwan Normal University, Taipei, Taiwan

**Keywords:** Confirmatory factor analysis, learning self-efficacy, medical education, self-efficacy, structural equation modeling

## Abstract

Learning self-efficacy (SE) assesses how learners understand and evaluate their ability to polish their learning process. Learning clinical medicine requires prolonged training, traditionally premised on longitudinal immersion in patient care. Such a process is domain-specific, whereas learning SE for clinical education remains under-explored. Unidimensional assessment is insufficient for capturing the inherent capabilities upon which well-trained physicians provide care. We aimed to establish a multi-dimensional learning SE questionnaire for clinical education among undergraduate medical students, evaluating the structure validity, followed by assessing the dimensionality of different models. Medical students of 2^nd^ to 4^th^ grades from Taiwan in 2022–2023 completed a multi-dimensional medical learning SE (MLSE) questionnaire, including four factors for basic science learning (conceptual understanding (CU), higher-order cognitive skills (HC), practical work (PW), and everyday application (EA)), and three for clinical mastery performance (medical communication (MC), evidence-based medicine (EBM), and Professionalism)). We tested factors’ intercorrelation, used exploratory and confirmatory factor analysis (EFA/CFA) for structure and validity assessment, and compared the fitness and dimensionality between models. Twenty-four items grouped into seven independent factors (3, 3, 4, 3, 5, 3, and 3 items in CU, HC, PW, EA, MC, EBM, and Professionalism, respectively) were established and finalized, with sufficient fitness, good convergent and construct validities. All MLSE factors significantly correlated (0.49–0.87; *p* < 0.001), demonstrating good convergent and discriminant validity. We established six models (first-order uncorrelated or correlated construct, one to three second-order dimensions (‘basic medical SE’, ‘clinical medical SE’, ‘Cognition’, or ‘Application’ of different structures), and a final model 7 containing four second-order dimensions (Cognition, Application, MC, and clinical medical SE) exhibiting adequate model fitness and measured learning SE satisfactorily. Our MLSE model structure disclosed vital SE factors with intercorrelations associated with medical students’ learning processes during clinical education. Polishing these dimensions may help promote their learning SE.

## Introduction

Self-efficacy describes the construct portraying how an individual understands and evaluates his/her ability to accomplish a context-specific task successfully. Proposed by Bandura more than 3 decades ago [1], self-efficacy attempts to emphasize one’s perception of one’s capacities to learn or perform behaviors, and promoting self-efficacy potentially assists one in improving performance. The concept of self-efficacy receives widespread attention following the introduction of the social cognitive theory, which dictates that social environment plays a key role in shaping individuals’ learning process and behaviors, and they can be motivated to act according to their beliefs regarding their abilities [2]. Bandura further demonstrated that self-efficacy and its belief modified our behavior by governing what we chose to do, how much effort we would devote, and how we persisted in completing the task when facing challenges [3].

Self-efficacy can be instrumental for learners, as those who believe in their capability are more likely to actively engage in learning and outperform others when encountering difficulties [4]. Students can rate their self-efficacy based on information gathered from their observations of role models, peers, environment, tutors’ persuasions, and their performances. Learners’ behaviors and perceptions of themselves may have stronger influences relative to experiences from vicarious sources [5]. The sense of self-efficacy toward learning contributes to students’ learning efficacy by motivating them to improve competence. Factors outside of (environmental) and inherent to learners (cognitive, biological, or affective) influence self-efficacy. Importantly, self-efficacy can be enhanced by positive feedback, favorable persuasions, and psychological cues [6].

Educators are increasingly interested in understanding how self-efficacy correlates with learning and development in medical schools [7]. Self-efficacy and its belief can assist medical students in improving their academic performances and achievement [8]. Motivation is also an integral element for facilitating medical students’ commitment to knowledge retention and clinical training, and greater motivation can form a positive cycle with dedicated learning behaviors toward self-regulated learning [9]. Higher self-efficacy leads to an increased likelihood of adopting deep strategic learning instead of a superficial one among medical students [10]. In addition, recognizing one’s self-efficacy may equip medical students to finish medical school studies successfully, help them face academic challenges, and make proactive career choices smoothly [11]. Medical students with better self-efficacy tend to believe that tasks are less tough than they appear and may be less likely to suffer from stress and mental suffering.

Learning self-efficacy refers to the perception of and confidence in how learners achieve their learning goals in a specific learning context and process [12]. This concept was similarly founded based on Bandura’s Social Cognitive Theory, focusing on learners’ determination regarding how they optimize their learning (the task) through effort investment, challenge handling, and perseverance during learning difficulty. Prior studies disclosed that learners with better learning self-efficacy could persevere longer during given courses and accomplish learning goals more readily after making choices [13]. The assessment of students’ learning self-efficacy can successfully predict their grades; Lane et al. demonstrated that learning self-efficacy was responsible for more than 10% of performance variance among postgraduate students [14]. Others similarly showed that greater learning self-efficacy helped students progress toward success in an online environment [15]. Importantly, students’ engagement status, learning profiles, and passivity can influence their learning self-efficacy and, potentially, academic performance [16]. These findings support the notion that learning self-efficacy dictates one’s future performance and can be modifiable through appropriate strategies.

Despite the importance of learning self-efficacy, relatively few studies address its role in medical students, especially in learning clinical medicine. While self-efficacy is a universal construct crucial for successful learning across all higher education contexts, medical students uniquely confront specific educational challenges that underscore the particular importance of investigating their learning self-efficacy [17]. Medical training is characterized by a prolonged, intensive educational process that integrates theoretical knowledge, practical skills, and professional attributes. Medical students must consistently apply foundational sciences to complex clinical scenarios, engage proficiently in evidence-based medicine, master effective medical communication with diverse patient populations, and uphold high professional and ethical standards. These demanding educational tasks and professional expectations create distinctive sources of stress, making medical students’ self-efficacy critical for their academic success, resilience, mental health, and future professional competence [18]. Therefore, examining learning self-efficacy within this context not only deepens understanding specific to medical education but also guides targeted educational interventions uniquely tailored for medical trainees.

Learning clinical medicine, or clinical education, is traditionally based on the longitudinal immersion in clinical patient care established decades ago, assuming medical students grow and mature after amassing experiences [19]. According to McGaghie et al.’s mastery learning theory, medical students and residents can be empowered and motivated for learning clinical medicine through a dedicated set of curricula and assessments encompassing essential skills, knowledge, and professional attributes, without time limitations for learners [19,20]. Within this framework, steps including baseline assessment, unit-based learning objectives and educational activity participation, standard setting and formative tests, followed by advancement to units of increasing difficulty, constitute the fundamental building blocks. However, the scope of this so-called baseline assessment remains poorly defined for clinical medicine learning. Apart from discipline-specific knowledge, it is widely acknowledged that evidence-based medicine and practice [21], medical professionalism [22], and multiple elements of physician-patient communication [23] should be infused into undergraduate medical education to better prepare students for clinical practice.

In addition, there is a need to integrate basic sciences into clinical education to facilitate clinical reasoning skills and develop critical decision-making [24]. Better conceptual understanding and fewer misconceptions have been shown to be an effective way for long-term scientific knowledge retention [25], assisting in subsequent clinical application. Cognitive skill training, from the sensory register and working memory to long-term storage, can contribute significantly to learning basic science and potentially to subsequent performance of complex tasks such as medical reasoning and decision-making [26]. The practicality of basic science knowledge for clinical application also helps elevate medical students’ motivation in learning and potentially provides them with confidence in clinical care [27]. Therefore, how medical students learn fundamental sciences may potentially influence how they take up clinical education at a later time as well.

Based on the above arguments, establishing a multi-dimensional instrument for assessing learning self-efficacy toward clinical education is an important issue that warrants serious attention. Current literature fails to construct a learning self-efficacy instrument for medical students that aims toward clinical education, necessitating considering its multi-dimensional nature. Well-educated medical students transitioning into clinical practice are expected to have various attributes that adapt themselves to the changing field of medicine, besides their core clinical competencies [28]. This constitutes an important knowledge gap regarding clinical medicine learning among medical students.

## Detailed objectives of this study

Building upon our prior research on learning self-efficacy, the current study aimed to construct and rigorously validate a multidimensional Medical Learning Self-Efficacy (MLSE) questionnaire specifically targeting undergraduate medical students. To achieve this overarching goal, we set out the following detailed objectives:

1. **Theory-driven item development**: To develop questionnaire items explicitly based on self-efficacy theory and competencies essential to clinical medicine, including evidence-based medicine, medical communication, and professionalism.
2. **Content validity, internal structure validation, and factor confirmation**: To ensure items’ clarity and appropriateness through literature review, expert panel validation, exploratory factor analysis (EFA) and confirmatory factor analysis (CFA) with responses collected.
3. **Reliability and validity assessment**: The internal consistency reliability of the questionnaire factors was evaluated using appropriate statistical indicators. Convergent and discriminant validity were examined by analyzing the correlations among the MLSE factors.
4. **Model comparisons**: To statistically compare multiple theoretically plausible competing models to determine the best-fitting model for accurately capturing medical students’ learning self-efficacy in clinical education.

## Materials and methods

### Ethical statement

The protocol of this study belongs to a parent study that has been approved by the institutional review board of National Taiwan University Hospital (NO. 202112011RINC). All participants provided verbal informed consent, and the study procedure complied with the Declaration of Helsinki.

### Enrollment of study participants

All participants in this study were medical students in their 2^nd^ to 4^th^ year at the National Taiwan University College of Medicine (NTU-CM) during 2022–2023. Part of the context during which we identified and recruited participants has been reported previously [29,30]. The selection of medical students specifically from 2^nd^ to 4^th^ grades was purposeful and based on our medical school’s curriculum structure. At the NTU-CM, the Small Group Tutorial (SGT) curriculum is explicitly designed to integrate basic sciences with clinical medicine for 2^nd^ to 4^th^-grade students, providing a coherent and relevant educational context ideal for exploring medical learning self-efficacy comprehensively. In contrast, students from the 1^st^ grade focus primarily on general education courses with limited clinical relevance, whereas students from 5^th^ and 6^th^ grades predominantly engage in full-time clinical rotations and internships, which differ significantly in educational context and learning expectations. Thus, we intentionally limited our sample to 2^nd^ to 4^th^-grade students to enhance the internal validity and coherence of the study context. Enrolled participants were mostly between 19 and 22 years old, with higher ages corresponding with higher grades.

### Inclusion and exclusion criteria

The inclusion criteria were clearly defined as: (1) medical students enrolled from the 2^nd^ to 4^th^ grade in NTU-CM between 2022 and 2023, and (2) participation in the SGT curriculum integrating basic and clinical medicine. Exclusion criteria included: (1) unwillingness or inability to provide informed consent, and (2) incomplete questionnaire responses at either measurement point.

After obtaining consent, participants were separatedinto two groups, each completing the MLSE questionnaire at a single time point. One group (*N* = 286) completed the MLSE at the beginning of one semester (used for EFA), and another group (*N* = 316) completed it at the end of the semester (used for CFA). These two groups were not formed through random assignment or stratification by grade level. Instead, they emerged naturally based on the timing of questionnaire administration during academic semesters. The different timings of questionnaire delivery were chosen to reduce the administrative burden.

### Sample size adequacy calculations

Sample size adequacy was guided by established recommendations in factor analytic methodology, suggesting a minimum subject-to-item ratio of approximately 5:1 to 10:1 [31,32]. Our finalized questionnaire contained 24 items and required a minimum target of 240 participants. The number of samplings, 286 for EFA and 316 for CFA, comfortably exceeded this threshold, ensuring adequate statistical power and reliable factor structure validation.

### Measurement theory and scale development procedure

We rigorously developed the MLSE questionnaire following well-established measurement theory guidelines [33,34]. Our development procedure consisted of four stages: (1) initial item generation grounded in the Social Cognitive Theory [12], prior validated scales of learning self-efficacy constructed accordingly [35], and essential competencies identified from clinical medicine literature (medical communication, evidence-based medicine, professionalism); (2) content validation by an expert panel comprising 11 medical educators and educational researchers, where each item was independently rated for clarity and relevance, and we retained items based on the content validity index (CVI) (item-level CVI, I-CVI ≥0.8); (3) EFA to identify the underlying factor structure; and (4) CFA to confirm the dimensionality and validate the factor structure. This structured and theory-based process ensured the methodological robustness of our instrument.

### MLSE development – the part of the basic science learning self-efficacy assessment

Based on Bandura’s Social Cognitive Theory [12], learning self-efficacy involves learners’ confidence and beliefs regarding their capabilities to perform learning tasks successfully. To ensure theoretical consistency, we adapted and rephrased existing validated items from a science learning self-efficacy scale [35], carefully integrating these with medical education-specific competencies identified in literature, including conceptual understanding (CU), higher-order cognitive skills (HC), practical work (PW), everyday application (EA), medical communication (MC), evidence-based medicine (EBM), and professionalism.

Measurement of self-efficacy can be broader at a generalized level or contain a high degree of specificity for meeting task requirements. The former approach exemplifies itself in several general self-efficacy scales that aim to assess one’s competence in tackling with novel tasks and coping with difficulties in diverse scenarios and across cultures [36,37]; however, Bandura himself has cautioned the researchers that self-efficacy measurements should be directed toward the specific judgment of capability for the realms of activities, levels of tasks, and under the specific circumstances [12]. Therefore, the latter approach, which is more context-specific, would be a better choice. There are several existing instruments for measuring learning self-efficacy for science, tested among primary school [38], high school [39], and university students [40]. Still, none involves scientific disciplines such as clinical medicine, which requires a vast array of professional knowledge basis, interpretation and application, skill honing, continuous practice, and self-improvement. Few tested the self-efficacy instrument in medical students. Furthermore, similar studies focus on clinical skill learning and cultivation only [41] without addressing clinical medicine as the entire learning spectrum. A more dedicated and scope-aligning instrument would be needed to increase the accuracy of our measurement.

Therefore, in this study, an MLSE questionnaire was developed to evaluate the multi-dimensional nature of learning SE in medical students, including dimensions and items derived and modified from the combination of a science learning self-efficacy instrument established previously [35,42] and clinical mastery performance development. The original science learning self-efficacy instrument was created based on well-established dimensions of self-efficacy in science education, including comprehension of related knowledge and skills, advanced cognitive techniques, practical work in science, and routine application of scientific concepts [43]. We included these dimensions to accommodate basic science learning, including CU, HC, PW, and EA, with 5, 5, 6, and 6 items in each dimension, respectively. For CU, medical students rated their belief in understanding the materials of various medical topics. A sample item of CU is ‘I can clearly describe medicine-related knowledge to others.’ For HC, medical students estimated their confidence in harnessing advanced cognitive strategies to think critically and solve learning problems. A sample item of HC is ‘I can critically evaluate various methods for solving medical problems.’ For PW, medical students considered their ability and competence to use common medical equipment well. A sample item of PW is ‘I can interpret the data and information of medical records.’ For EA, medical students addressed their confidence in applying concepts routinely to their practices. A sample item of EA is ‘I can use relevant medical concepts to explain many phenomena in daily life.’

We further introduced a dimension, ‘medical communication’ (MC), with five items, to emphasize the importance of communication for health professionals, which aims to optimize physician–patient relationships and improve patients’ quality of life [44]. The construct of these five dimensions of medical self-efficacy (CU, HC, PW, EA, and MC) has been tested previously in a post-graduate setting among health professionals, whose results disclosed that all items of each dimension exhibited significant loadings ( > 0.7) [45]. The Cronbach’s alpha of the five dimensions ranged between 0.85 and 0.94, and the construct reliability of the original MLSE fell between 0.80 and 0.95. The construct had an excellent goodness of fit value in the validation study [45]. A sample item of MC is ‘I can clearly explain the medical knowledge I have learned to others.’

### MLSE development – the part of the clinical mastery performance learning self-efficacy assessment (EBM, professionalism, and internet self-efficacy)

To enrich its multi-dimensional nature and to better adapt the content to undergraduate medical students, we initially considered another three independent dimensions, EBM, Professionalism, and Internet self-efficacy, based on a medical education literature review [46,47] and input from experts in our institute.

EBM entails using specific scientific methods to organize, summarize, and apply data to inform healthcare decisions [48]. Existing studies disclose that EBM education significantly improves medical-allied students’ self-efficacy and increases their clinical competence [49]. A qualitative study reveals that EBM assists medical students in problem-solving, sharpens their thinking, and enhances confidence [50]. We allocated five items to this dimension based on the EBM principles outlined previously [51]. Professionalism denotes the attitudes, stands, and/or values and the commitment to uphold integrity, express empathy and compassion, and foster appropriate patient relationships as a health professional [52]. It is a core quality that underlies the moral binding between health professionals and patients and has been defined by the American Board of Internal Medicine to include multiple attributes/merits (e.g., accountability, altruism, duty, excellence, integrity, and respect for patient autonomy) [53]. It has been proposed that professionalism be incorporated into undergraduate medical education as early as possible and cultivated by providing multiple learning opportunities to improve clinical competence [54]. We designed five items assessing medical professionalism to reflect the importance of this dimension in learning, according to its core elements shown previously [55]. Internet self-efficacy describes an individual’s perception of his/her competency to fully harness the internet [47], and its importance in helping medical students adapt to the current online learning environment has been promptly appreciated.

An expert meeting was convened, including 11 members consisting of medical education researchers (with internal medicine, surgery, emergency medicine, or pediatrics specialties), the faculty from the graduate institute of medical education in our school, senior tutors of SGT and clinical medicine, and our medical school administrators, all with more than 5 years of relevant experiences. All questionnaire items were independently rated by these experts for clarity and appropriateness on a 4-point scale. We calculated the I-CVI, retaining only items with an I-CVI score ≥ 0.8 (range: 0.82 to 1.0), thus ensuring robust content validity and reducing potential arbitrariness. The consensus concluded that the two dimensions, EBM, whose competency and necessity are widely recognized in the real-world practice [48], and professionalism, whose role assumes rising importance in the evolving pattern of medical practices [52], should be incorporated into this new MLSE questionnaire. However, the Internet self-efficacy dimension is considered conceptually different from the other dimensions, making it difficult to align with the overall structure. Internet self-efficacy emphasizes digital abilities instead of focusing on professional skills in medicine-related SE. Moreover, Internet self-efficacy was deemed to belong to a separate domain of skills more related to general technology use but not medicine-related SE.

Moreover, we employed cognitive interviewing [56,57] to refine item wording, assess item comprehension, and confirm interpretative consistency among respondents. We conducted interviews with students, asking them to interpret each item and discuss their reasoning process verbally. This allowed identification and clarification of ambiguities, ensuring the finalized items accurately captured students’ interpretations and meanings, thus substantially improving content validity and the questionnaire’s overall reliability.

### MLSE development – assessing the combination of the basic science and clinical mastery performance dimensions

The content of our MLSE finally contained seven dimensions (CU, HC, PW, EA, MC, EBM, and Professionalism) and 37 items (5, 5, 6, 6, 5, 5, and 5 items for each dimension, respectively). Each item was rated using a 5-point Likert-type scale, from 1 (strongly disagree) to 5 (strongly agree). This questionnaire subsequently underwent a focused discussion by the same panel of experienced medical education experts described above in NTU-CM, with optimized wordings and phrases. We used CVI to ensure the validity of our MLSE instrument by examining the degree to which item ingredients fit the designated dimension.

### Statistical analysis

Analyses used in this study were done using the SPSS 25.0 software and AMOS 21.0. We tested the dimension validity of our MLSE scale using EFA and then CFA. To rigorously establish the factor structure and validity of the MLSE, we first conducted an EFA by using principal component factoring and varimax rotation methods according to established methodological criteria [32,58], retaining items with factor loadings ≥ 0.40 on their designated factor and less than 0.40 on all other factors. Subsequently, we conducted CFA with the Maximum Likelihood (ML) estimation method following the guidelines proposed by Kline [59] and Hu & Bentler [60]. Model fit was evaluated using multiple indices, including χ^2^/df < 5.0, RMSEA < 0.08, SRMR < 0.08, and GFI, NFI, IFI, TLI, and CFI values ≥ 0.90 [32,59,60]. These stringent criteria ensured that our MLSE’s final structure was methodologically sound and robust. In addition to Cronbach’s alpha, we computed McDonald’s omega (ω) to estimate reliability more accurately, given its suitability for multidimensional scales [61]. We also evaluated the normality of each MLSE factor based on the corresponding skewness and kurtosis values.

Next, we examined the associations between factors using Pearson’s correlation since intercorrelation coefficients among factors in the questionnaire are crucial for evaluating the discriminant validity of constructs. The discriminant validity was assessed based on the criterion proposed by Fornell & Larcker [62], which requires the square root of each factor’s average variance extracted (AVE) to exceed its inter-factor correlations. Finally, we compared the fitness and the dimensionality of different suggested models originating from CFA results and provided the fitness matrices using structural equation modeling. We visualized each conceptualization model in figures.

## Results

### Exploratory factor analysis (EFA) results

Among 413 students, the final response rate was 69.2%. Our EFA analyses, incorporating responses from 286 medical students (mean age 21.3 ± 0.2 years old, 77.6% male), showed that 24 items were retained and grouped into seven factors, including CU (3 items; factor loadings: 0.60–0.68), HC (3 items; factor loadings: 0.54–0.67), PW (4 items; factor loadings: 0.82–0.89), EA (3 items; factor loadings: 0.59–0.73), MC (5 items; factor loadings: 0.73–0.79), EBM (3 items; factor loadings: 0.58–0.81) and Professionalism (3 items; factor loadings: 0.78–0.82). The results in Table 1 show that the factor loadings were all higher than 0.50 (between 0.54 and 0.89), and the total variance explained was 83.24%. The Cronbach’s alpha values (reliability coefficient) for each factor ranged from 0.85 to 0.96, and the total alpha value was 0.96. The McDonald’s omega (ω) coefficient for each factor was acceptable (ω = 0.85 to 0.96), and the total omega value also showed high reliability (ω = .96). The EFA results showed satisfactory internal reliability and validity for further CFA.

**Table 1.** The EFA analysis of medical learning self-efficacy (MLSE) (*N* = 286).

Factor	Item numbers	Factor loading		Cronbach’s alpha value	McDonald’s omega (ω) coefficient
		Min	Max		
Conceptual understanding	3	0.60	0.68	0.85	0.85
Higher-order cognitive skills	3	0.54	0.67	0.93	0.96
Practical work	4	0.82	0.89	0.96	0.88
Everyday application	3	0.59	0.73	0.87	0.90
Medical communication	5	0.73	0.79	0.93	0.93
Evidence-based medicine	3	0.58	0.81	0.87	0.88
Professionalism	3	0.78	0.82	0.90	0.90

Total alpha value:0.96; total omega value: 0.96.

### Confirmatory factor analysis (CFA) results

According to the EFA results with the items selected, we then conducted CFA to measure the MLSE’s construct validity and reliability using the responses collected from 316 medical students (mean age 21.4 ± 2.6 years old, 75.3% male) in the other semester. No error terms were allowed to correlate in our CFA model, as no theoretical justification warranted correlating residuals. Each factor’s items were assumed to independently reflect the latent construct. Based on and parallel to the EFA results, CFA finalized the MLSE with 24 items in the final model (i.e., 3, 3, 4, 3, 5, 3, and 3 items in CU, HC, PW, EA, MC, EBM, and Professionalism factors, respectively). According to Kline [59], absolute skewness values less than 3 and kurtosis values less than 10 indicate normal distribution. The skewness (ranging from −0.96 to −0.31) and kurtosis (ranging from −0.71 to 2.17) values for each factor of the MLSE all fell within these acceptable ranges, supporting the assumption of normality.

Table 2 shows the CFA results for the MLSE in one single model with factor loadings, AVE, composite reliability (CR), Cronbach’s alpha values, and McDonald’s omega coefficient value, as well as the mean and standard deviation (SD) for each factor presented. All the items’ factor loadings were greater than 0.50 (between 0.80 and 0.92). The AVE values were all above 0.50 (between 0.70 and 0.85), the CR values exceeded 0.70 (0.88–0.96), the Cronbach’s alpha values were higher than 0.70 (0.88–0.94), and the McDonald’s omega coefficient values [61] were higher than 0.70 (0.88–0.96), suggesting that our CFA model can be validated as being acceptable [32,62]. Regarding the goodness of fit, χ^2^/df 3.57, RMSEA 0.090, SRMR 0.040, GFI 0.81, NFI 0.90, IFI 0.93, TLI 0.91, and CFI 0.93, the fit scale for this model, approaching the criterion of a good fit, showed a sufficient fit [59,60,63–65]. However, the GFI in this model is modestly lower than 0.90; prior studies suggested that 0.80 was the cut-off value indicating moderate acceptability [66]. In addition, the RMSEA of our MLSE fell between 0.08 and 0.10, supporting an acceptable fit [64,67]. Consequently, our CFA results confirmed both the convergent and construct validities for the MLSE. The final CFA confirmed a seven-dimensional factor structure of the MLSE scale, comprising the following dimensions: Conceptual Understanding, Higher-order cognitive skills, Practical work, Everyday application, Medical communication, Evidence-based Medicine, and Professionalism. Thus, the final confirmed MLSE scale robustly captures the multidimensional nature of medical learning self-efficacy.

**Table 2.** The CFA analysis of medical learning self-efficacy (MLSE) (*N* = 316).

Factor and item	Factor loading	*t*-value	AVE	CR	Cronbach’s alpha value	McDonald’s omega (ω) coefficient	Mean	*SD*
**Conceptual understanding (CU)**	–	–	0.73	0.89	0.89	0.89	3.83	0.75
CU 1	0.89	–						
CU 2	0.86	20.71*						
CU 3	0.82	18.96*						
**Higher-order cognitive skills (HC)**	–	–	0.77	0.91	0.90	0.91	3.63	0.89
HC 1	0.87	–						
HC 2	0.85	20.33*						
HC 3	0.91	23.33*						
**Practical work (PW)**	–	–	0.85	0.96	0.96	0.96	3.13	1.13
PW 1	0.92	–						
PW 2	0.95	31.30*						
PW 3	0.89	26.05*						
PW 4	0.92	28.52*						
**Everyday application (EA)**	–	–	0.72	0.89	0.88	0.89	3.65	0.83
EA 1	0.87	–						
EA 2	0.80	18.15*						
EA 3	0.88	21.45*						
**Medical communication (MC)**	–	–	0.77	0.94	0.94	0.94	3.86	0.80
MC 1	0.86	–						
MC 2	0.86	20.46*						
MC 3	0.91	22.80*						
MC 4	0.87	20.76*						
MC 5	0.88	21.15*						
**Evidence-based medicine (EBM)**	–	–	0.75	0.90	0.90	0.90	3.48	0.95
EBM 1	0.85	–						
EBM 2	0.88	19.96*						
EBM 3	0.86	19.40*						
**Professionalism (Pro)**	–	–	0.70	0.88	0.88	0.88	3.81	0.79
Pro 1	0.85	–						
Pro 2	0.85	18.58*						
Pro 3	0.81	17.22*						

Total alpha value: 0.97; total omega value: 0.97.

CFA: Confirmatory factor analysis; AVE: Average variance extracted; CR: Composite reliability; SD: Standard deviation; *p < 0.001.

### Pearson’s inter-correlation and discriminant validity

Table 3 shows the inter-correlations between all the seven MLSE factors. All MLSE factors showed significant correlations, with coefficients ranging from 0.49 to 0.87 (p < 0.001). Hair et al. stated that intercorrelations above 0.90 might suggest redundancy or poor discriminant validity among factors [32]. In addition, the square roots of the AVE values for each factor (0.84–0.92) were larger than the correlation coefficients between all seven factors of the MLSE. These results indicate a good level of convergent and also discriminant validity for our MLSE.

**Table 3.** Inter-correlation between the seven factors of MLSE (*N* = 316).

Variables	1	2	3	4	5	6	7
1. Conceptual understanding	**(0.85)**						
2. Higher-order cognitive skills	0.80*	**(0.88)**					
3. Practical work	0.61*	0.73*	**(0.92)**				
4. Everyday application	0.73*	0.87*	0.73*	**(0.85)**			
5. Medical communication	0.77*	0.78*	0.49*	0.75*	**(0.88)**		
6. Evidence-based medicine	0.68*	0.77*	0.75*	0.74*	0.67*	**(0.87)**	
7. Professionalism	0.64*	0.72*	0.57*	0.72*	0.71*	0.74*	**(0.84)**

*p < 0.001 (two-tailed). The square roots of the AVE are shown by diagonal values (bold figures). Off-diagonal values show correlations between factors.

### Comparisons of the fit statistics of the proposed models

Because science learning self-efficacy is multifaceted [35], evaluating different models provides a better comprehension of the relationships between the questionnaire items and factors [68]. Six distinct models were therefore proposed, developed, and subject to evaluation to help explain the relationships between the seven MLSE factors.

Figures 1a–1f outlines the six original proposed models for our MLSE. Model 1 described an uncorrelated construct that examined whether the seven factors in our MLSE are independent. Model 2 further presented the correlated model, which examined whether the seven factors in the MLSE are mutually correlated. In Model 3, a Basic MSE (medical self-efficacy), according to the science and nursing learning SE construct reported previously [40,69], was designed as the second-order dimension consisting of five factors: CU, HC, PW, EA, and MC; the Basic MSE dimension correlated with the two first-order factors: EBM and Professionalism. Following Model 3, Model 4 introduced two intercorrelated second-order dimensions, Basic MSE and a new clinically oriented dimension, Clinical MSE, which consists of two factors: EBM and Professionalism. In Model 5, we moved the MC factor to the second-order Clinical MSE dimension, as medical communication can also be an important property for clinical practice. In Model 6, we proposed three intercorrelated dimensions that differed from the Model 5 design. Based on the findings from other studies [35,69], Cognition was introduced as a second-order dimension consisting of two factors: CU and HC. Application was also added as a second-order dimension consisting of three factors: PW, EA, and MC. Meanwhile, Clinical MSE was assigned as the third second-order dimension with two factors: EBM and Professionalism. In particular, the three second-order dimensions (Cognition, Application, and Clinical MSE) were set to be correlated.

**Figure 1a.**
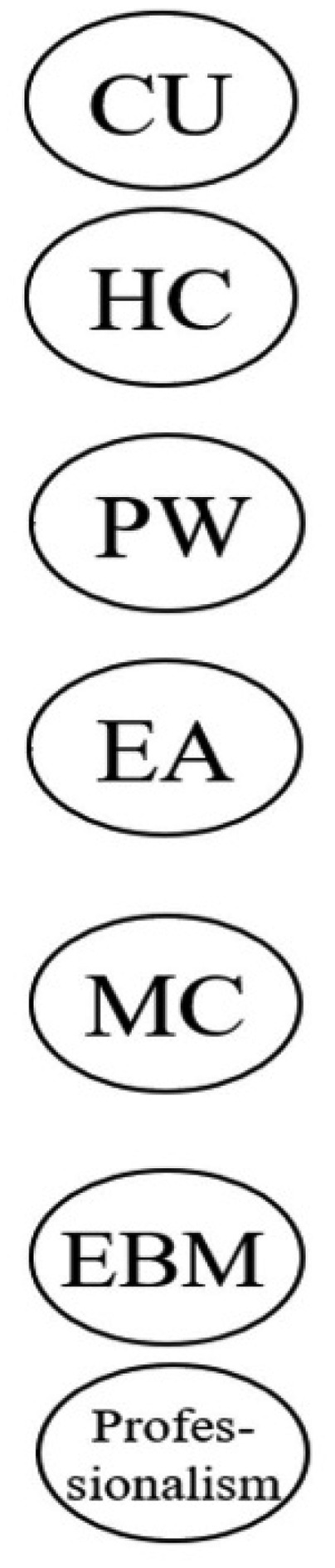
Model 1 (uncorrelated first-order model): all seven MLSE factors (CU, HC, PW, EA, MC, EBM, and professionalism) are modeled as independent first-order constructs with no correlations or higher-order grouping.

**Figure 1b.**
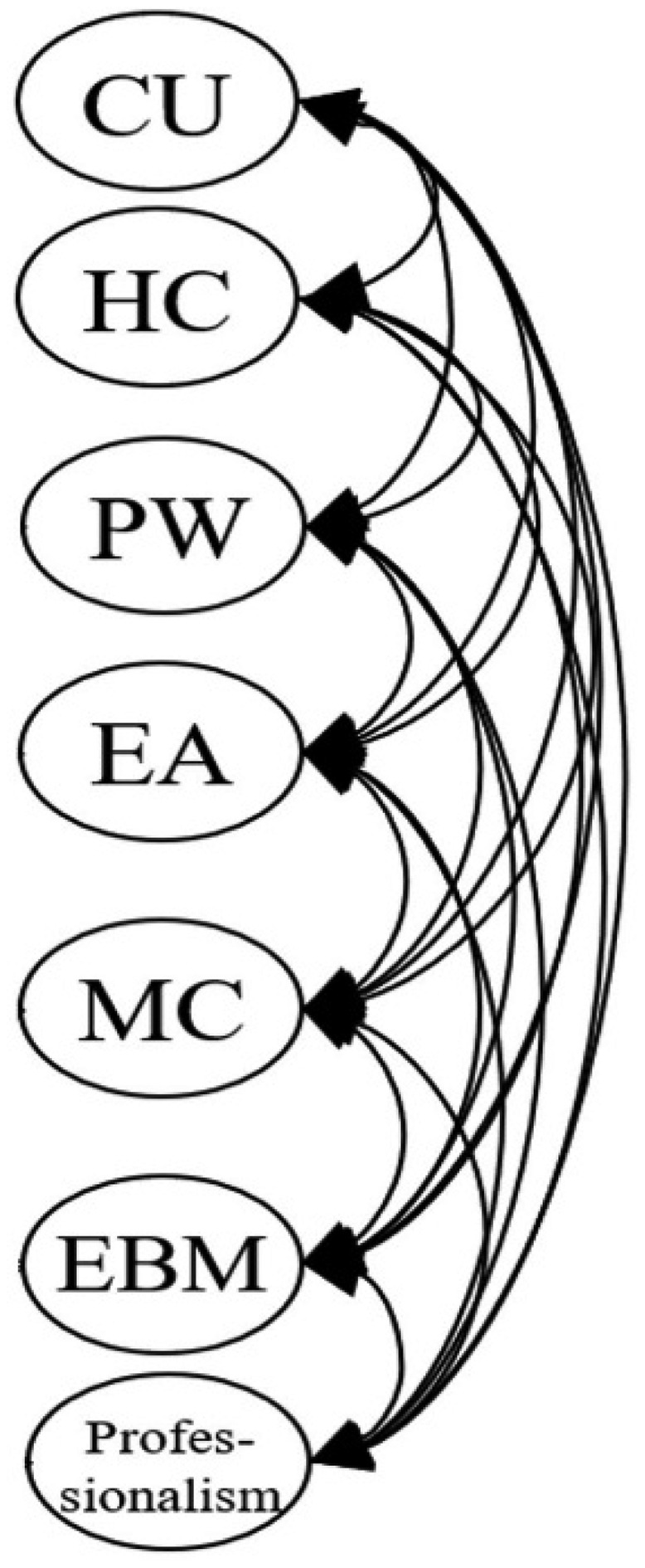
Model 2 (correlated first-order model): all seven first-order MLSE factors (CU, HC, PW, EA, MC, EBM, and professionalism) are modeled with free correlations among them. No second-order constructs are specified.

**Figure 1c.**
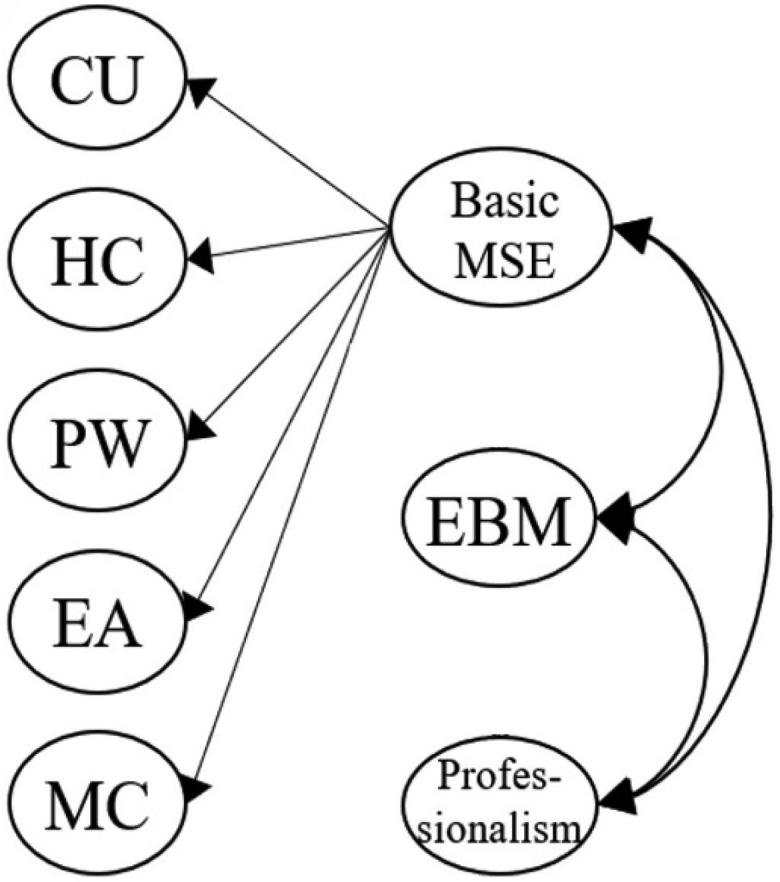
Model 3 (one second-order construct, *basic MSE*): five first-order factors (CU, HC, PW, EA, and MC) are grouped under a single second-order factor, referred to as basic MSE. EBM and Professionalism remain as independent first-order factors.

**Figure 1d.**
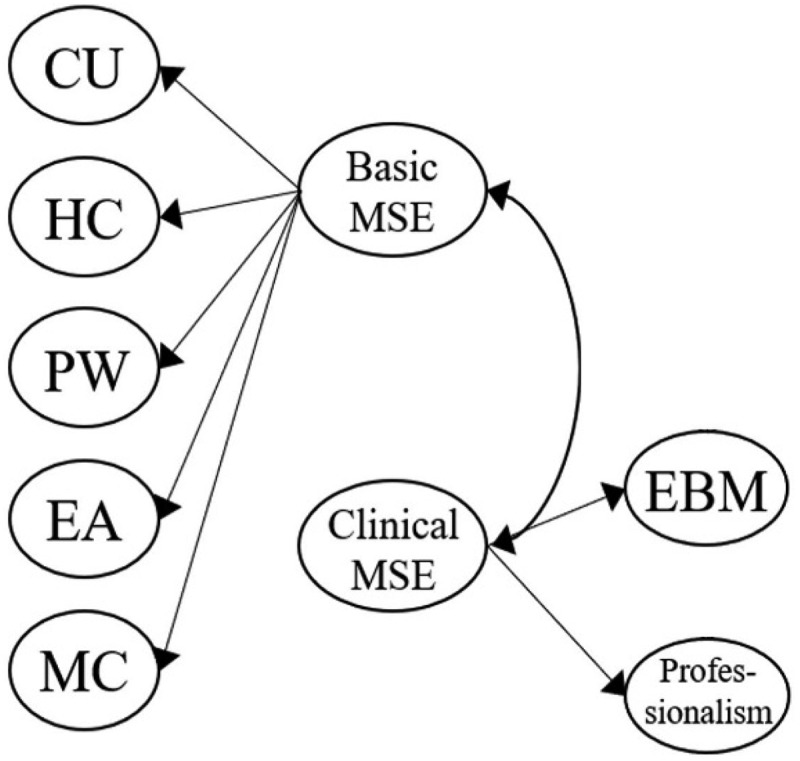
Model 4 (two second-order constructs, *basic MSE* and *clinical MSE*): CU, HC, PW, EA, and MC are grouped as first-order factors under basic MSE. EBM and Professionalism are grouped as first-order factors under a second-order factor labeled clinical MSE. Two second-order factors (basic MSE and Clinical MSE) are specified.

**Figure 1e.**
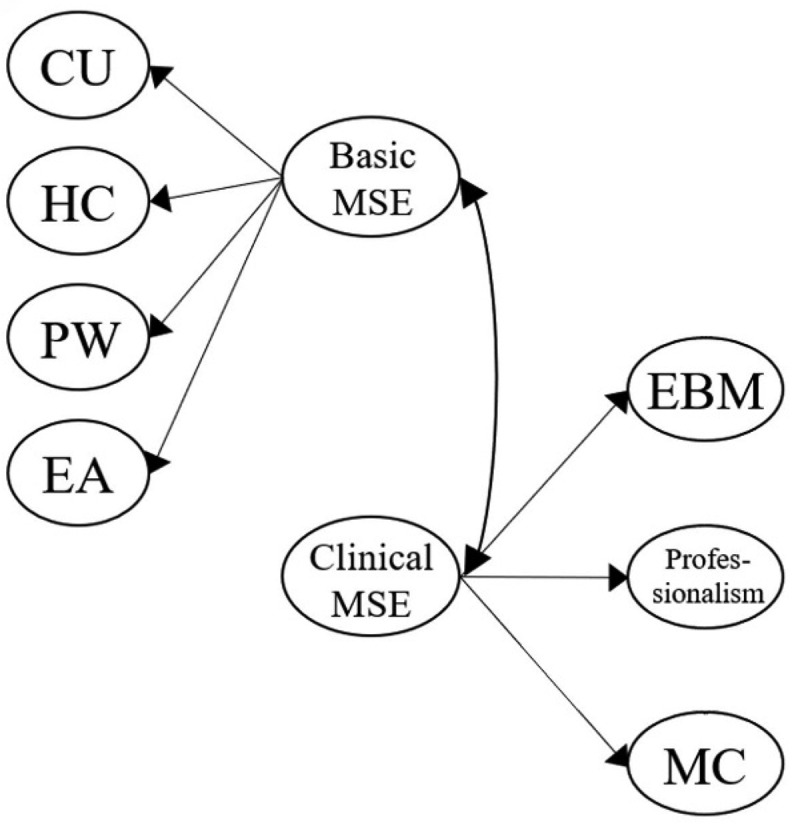
Model 5 (reassignment of MC under clinical MSE): In contrast to model 4, MC is reassigned as a first-order factor under clinical MSE, along with EBM and Professionalism. CU, HC, PW, and EA are grouped as first-order factors under basic MSE. Thus, both basic MSE and Clinical MSE serve as second-order constructs.

**Figure 1f.**
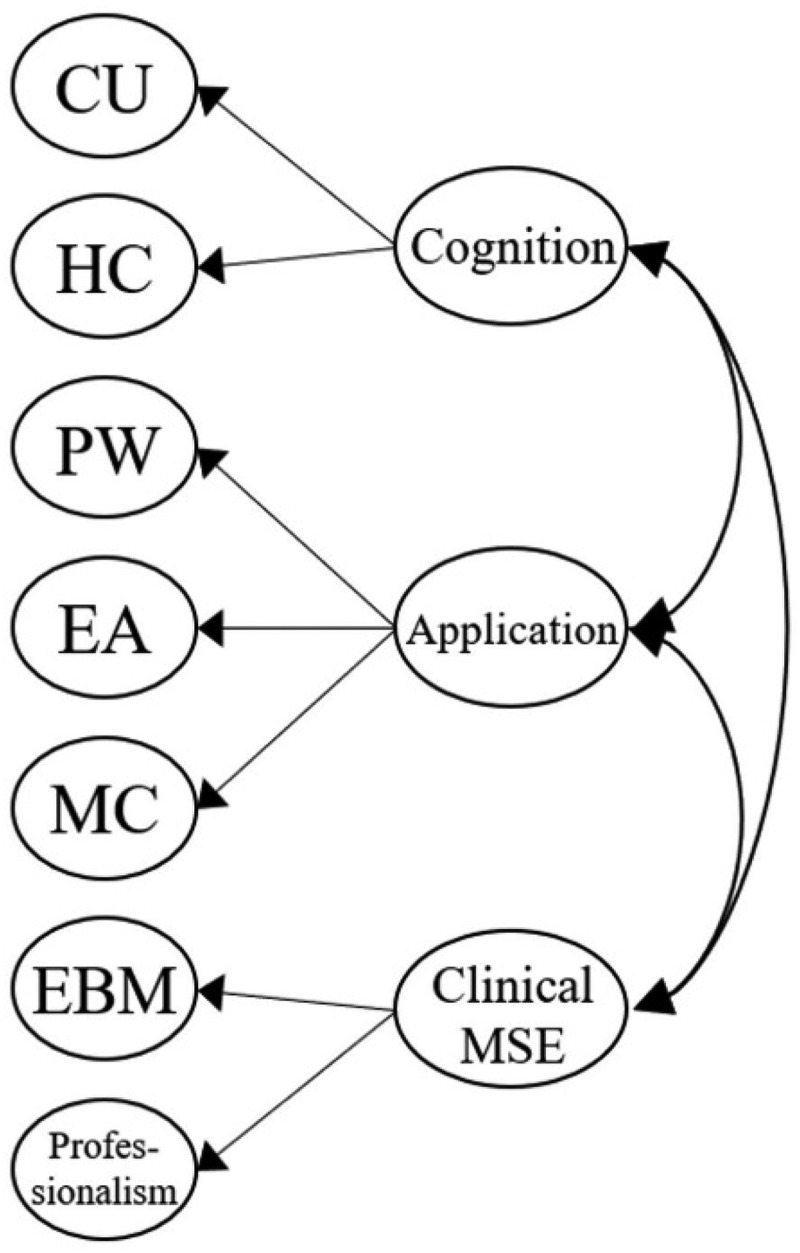
Model 6 (three second-order constructs, *Cognition*, *Application*, and *clinical MSE*): CU and HC are modeled as first-order factors under ‘Cognition.’ PW, EA, and MC are under ‘application.’ EBM and Professionalism are under ‘clinical MSE.’ three second-order constructs (cognition, application, clinical MSE) are defined.

We categorized Models 1 and 2 as the first-order models, whereas Models 3 to 6 were classified as the second-order models. The fit indices results of all models are shown in Table 4.

**Table 4.** Fit indices of the seven proposed models in MLSE.

Model	χ^2^/df	RMSEA	SRMR	GFI	NFI	IFI	TLI	CFI
Model 1	11.50	0.183	0.532	0.48	0.66	0.68	0.65	0.68
Model 2	3.57	0.090	0.040	0.81	0.90	0.93	0.91	0.93
Model 3	3.88	0.096	0.058	0.80	0.89	0.92	0.91	0.92
Model 4	3.88	0.096	0.058	0.80	0.89	0.92	0.91	0.92
Model 5	3.90	0.096	0.058	0.80	0.89	0.92	0.91	0.92
Model 6*	3.87	0.095	0.056	0.80	0.89	0.92	0.91	0.92
Model 7	3.82	0.095	0.055	0.81	0.90	0.92	0.91	0.92
Suggested values	<5.0	<0.08	<0.08	>0.90	>0.90	>0.90	>0.90	>0.90

^*^The model is not positively defined.

### The first-order models

Model 1 tested whether the assumption that all seven factors of MLSE were independent and unrelated was valid. The fit indices of Model 1 (Table 4) indicated that it is an unacceptable model since Model 1 is not functional, but could serve as a baseline model for comparison with all the others by providing the ground information.

Model 2, a simple correlated model also known as the CFA model, showed that the MLSE could be clearly divided into seven distinct but linked factors with acceptable fit indices (χ^2^/df 3.57, RMSEA 0.090, SRMR 0.040, GFI 0.81, NFI 0.90, IFI 0.93, TLI 0.91, and CFI 0.93) (Table 4). These findings support the utility of this model when investigating the relationships between medical students’ multiple learning self-efficacy factors and various learning outcomes. A potential approach would be to use our MLSE’s individual factor(s) as the independent variable(s) to predict medical students’ learning outcomes (e.g., scores, grades, skills, or learning engagement).

### The second-order models

Studies have uncovered the multifaceted nature of learning SE [35,69], and comparing models with different constructs (e.g., first-ordered or second-ordered ones) allows for a better understanding of the designed items and structures of the developed instruments. In addition to Models 1 and 2 (first-order ones), we developed the other four second-order models, aiming to elucidate associations between the seven factors of our MLSE. These models included Models 3–6, which embedded the second-order dimension into the proposed construct. These designed models are detailed below.

Model 3 was designed as a one-factor second-order dimension model (Basic MSE) that correlated with the other two first-order factors (EBM and Professionalism), exhibiting acceptable fit indices (χ^2^/df 3.88, RMSEA 0.096, SRMR 0.058, GFI 0.80, NFI 0.89, IFI 0.92, TLI 0.91, and CFI 0.92) (Table 4). This model restructured the five factors described previously [35,69] into a higher-order dimension construct. Instead of using a complex design (e.g., Model 2), Model 3 might be better equipped to summarize the whole picture of the five basic medical SE of the medical students as one single composite variable. This model also provides insights to researchers interested in examining the relationship between basic MSE and the two first-order factors for medical students: EBM and Professionalism. Furthermore, compared to the relative complexity of Model 2, researchers may simply use Model 3’s three first- or second-order variables as the antecedents to predict medical students’ learning outcomes.

In Model 4, we grouped EBM and Professionalism into another second-order dimension, Clinical MSE, to examine its correlation with another second-order dimension, Basic MSE. Previous studies highlighted the role of communication skills during clinical practice and concluded that they are vital clinical skills [70]. In Model 5, the first-order factor MC was reassigned to the second-order dimension, Clinical MSE. Models 4 and 5 show acceptable fit indices (for Model 4, χ^2^/df 3.88, RMSEA 0.096, SRMR 0.058, GFI 0.80, NFI 0.89, IFI 0.92, TLI 0.91, and CFI 0.92; for Model 5, χ^2^/df 3.90, RMSEA 0.096, SRMR 0.058, GFI 0.80, NFI 0.89, IFI 0.92, TLI 0.91, and CFI 0.92) (Table 4). These two models simplified the seven first-order factors into only two second-order dimensions, Basic and Clinical MSE. Ho et al. previously revealed that students’ science learning SE could be predicted by their learning conceptions and self-regulated learning [71]. Therefore, Basic and Clinical MSEs may serve as the outcome variables for measuring the predictions of medical students’ learning variables (e.g., learning medical conceptions or self-regulated learning in learning medicine).

Model 6 was specifically designed as a three-factor second-order dimension model, consisting of Cognition (including first-order factors CU and HC), Application (including first-order factors PW, EA, and MC), and Clinical MSE (including first-order factors EBM and Professionalism). However, this model structure was not positively defined (Table 4), suggesting that Model 6 was not a qualified and fitting model for representing medical students’ medical learning SE. A modified model would be needed after refining Model 6.

**Figure 2.**
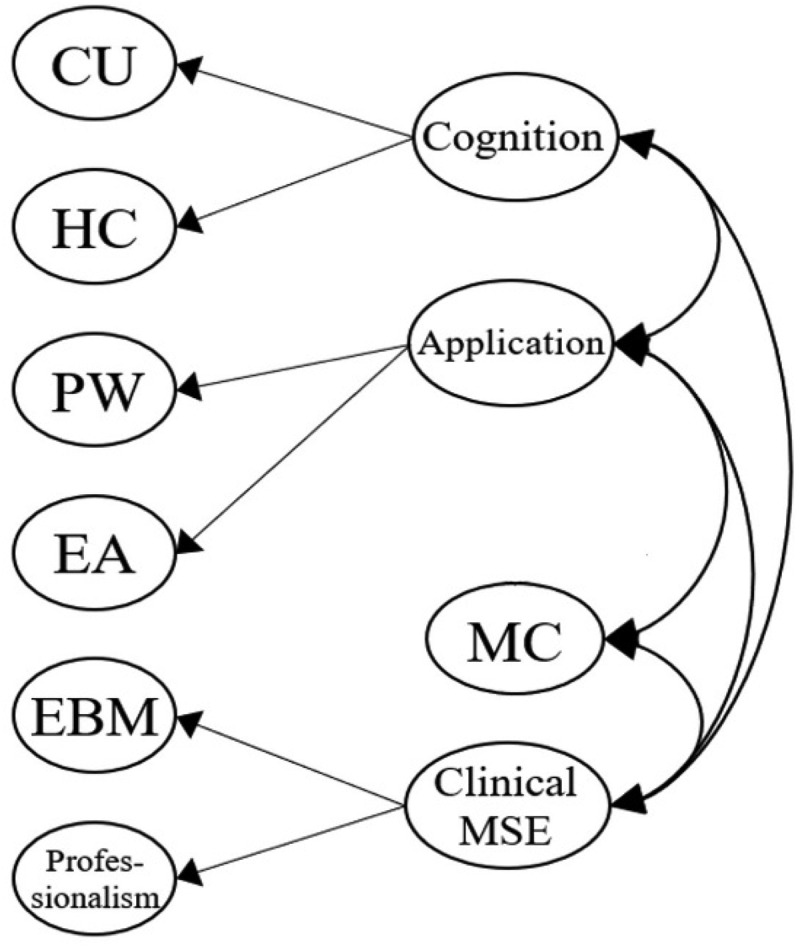
Model 7 (three second-order constructs with MC as a standalone first-order factor): CU and HC are first-order factors under “Cognition.” PW and EA are under “application.” EBM and Professionalism are under “clinical MSE.” MC remains as an independent first-order factor, correlated with all second-order constructs.

Others suggested that medical communication was a unique and independent factor in health profession education and should be isolated from other factors [69]. We, therefore, created Model 7 (Figure 2), in which MC was isolated as a single first-order factor and correlated with three second-order dimensions: Cognition, Application, and Clinical MSE. Model 7 showed acceptable fit indices results (χ^2^/df 3.82, RMSEA 0.095, SRMR 0.055, GFI 0.81, NFI 0.90, IFI 0.92, TLI 0.91, and CFI 0.92) (Table 4) and was positively defined. This alternatively designed Model 7 is presented as a qualified model for measuring medical students’ learning SE, with the advantages of combining vital dimensions (such as Cognition, Application, and Medical communication) with a newly introduced dimension, Clinical MSE.

## Discussion

In this study, we recruited medical students participating in a small group tutorial curriculum focusing on basic-to-clinical medical sciences and measured their medical learning SE using the MLSE questionnaire adapted and enriched based on prior studies [35,69]. The MLSE questionnaire had seven factors with 24 items and exhibited good construct, internal, and external validity. We further established different models aiming to explain the correlations between these seven factors; interestingly, we validated several dimensions that assisted us in understanding the structure of medical students’ MLSE, including basic and clinical MSE, cognition, and application. Future studies are welcomed to harness our model structure to test whether these dimensions correlate with medical students’ learning processes and outcomes and, more importantly, how we can polish these dimensions to promote medical students’ learning SE.

In this study, we confirmed that the seven factors were independent elements inherent to medical students’ learning SE. They were intercorrelated, as Model 1 was deemed unqualified, whereas Model 2 accrued fair fitness indices (Table 4). Existing studies have shown that the five original learning SE factors (CU, HC, PW, EA, and MC) can be simplified further into a second-order dimension [16,35,69], and this has also been affirmed in our models (Models 3 and 4). However, we further revealed that medical communication emerged as an instrumental factor that exhibited tight correlations with other second-order dimensions (basic and clinical MSE, cognition, and application), supporting its cross-dimensional role in medical learning (Figure 1). Factors including EBM and Professionalism exhibited independent correlations with the basic MSE (Model 3) and medical communication, and together, they could be simplified into clinical MSE (Models 4 and 7) (Figures 1 and 2).

It is interesting to find that Model 6, in which cognition and application dimensions were set in second-order scales, did not achieve adequate model fitness and was deemed unqualified (Table 4). Wang et al. previously demonstrated that these two dimensions were independent SE factors for science learning among university students [40], but this is not the case when incorporating medical communication together in our findings. When we singled out medical communication from the application dimension in Model 7, the model structure regained validity, indicating that medical communication might not belong to the application dimension found in previous studies [69]. In fact, medical communication has been described as ‘a chain of actions’ linking cognitive and empathic processes to influence the behaviors of clinicians and patients [72]. Such conceptualization potentially exemplifies the role of medical communication as the bridge between cognition and behavioral (or application) components of clinical practices. Indeed, medical students perceive that medical communication enables them to gather information for diagnosis and care provision from patients, both of which belong to the application dimension [73] but are not the entirety of clinical care itself. Medical students tend to value communication skills as a vital attribute of physicians, and such skills independently contribute to patients’ health and satisfaction [74].

Two important factors were introduced and applied to our medical learning SE, EBM and professionalism. EBM is a clinically oriented method that aims to assist clinicians in appropriate question formulation, securing the usefulness of information, and synthesizing available data for facilitating patient care. EBM has been popularized as a core curriculum in undergraduate medical education since decades ago [75], but few explore whether it can be an independent SE factor during learning medicine. A prior systematic review focusing on EBM training in undergraduate medical education found only 30% out of 20 studies targeted preclinical students and rarely addressed EBM as an independent attribute of learning SE [76]. In an EBM workshop report, Nieman et al. found that first-year medical students had their SE enhanced after the course, supporting the importance of EBM teaching in undergraduate medical education [77]. On the other hand, professionalism in medicine, the subordination of self-interest to patients as a primacy, has its roots in the Hippocrates era and remains constant as the ethical code of contemporary medicine [78]. Medical students’ understandings and perceptions about professionalism in medicine, however, are rather diverse; Byszewski et al. reported that undergraduate medical students described professionalism in actions/behaviors, including respect, integrity, honesty, compassion, dedication, and empathy [79]. On the contrary, to a wider range of stakeholders, professionalism in medicine may expand to include themes such as compliance with ethical values, self-audit, motivation, ensuring accessibility and care continuity, teamwork, and having appropriate manners [80]. The insufficient understanding of professionalism among medical students, contrary to the view of educators/doctors/allied health professionals, in combination with medical students’ pursuit of role models [79], influences their perception of the importance of professionalism in learning medicine. Despite this, very few reports address professionalism as an indispensable factor in learning SE among medical students. Our findings thus pave the way toward expanding the scope of medical learning SE. Improving EBM practice and upholding professionalism, therefore, carry the potential for promoting medical learning SE among undergraduate medical students.

Our results may have educational implications. Previous studies indicated that self-efficacy could serve as the predictor or mediator of learning efficacy or even be a distinct type of learning outcome [17,18,81]. Teachers should adopt strategies that aim to sharpen cognition and enrich the application dimension, which may be beneficial for improving medical students’ learning SE, including metacognitive training and coaching [82]. Moreover, medical students’ SE has been found to be positively correlated with evidence-based practice and training [83], supporting the latter’s potential in promoting learning SE. Emphasizing professionalism during undergraduate medical education through role model introduction and altruism enhancement may also lay the foundation for subsequent medical learning through establishing self-awareness and pride in the profession [84].

Our study has its strengths and limitations. Our participants represented pre-clinical medical students at our institution in terms of age, gender distribution, and academic year distribution. However, caution should be exercised when generalizing our findings to other institutions or medical students with different demographic characteristics. Due to constraints posed by our initial study design, several advanced psychometric analyses were not included, including measurement invariance testing, Item Response Theory (IRT) modeling, and test–retest reliability. Measurement invariance testing would be beneficial for assessing subgroup differences; IRT could further validate item-level functioning, particularly in larger-scale studies; and test–retest reliability would provide insights into scale stability. These represent valuable avenues for future research to validate and refine our MLSE instrument. Our study established and validated a new medical learning self-efficacy questionnaire consisting of seven factors, among which two (EBM and professionalism) were underappreciated. We identified important structures from different models built upon these factors and explained their complex relationship. However, limitations do exist. We did not measure all the potential interfering factors that might be associated with self-efficacy, such as motivation and interpersonal interactions. The educational context may also play a role in affecting learning self-efficacy. Our SGT curriculum contains materials spanning from basic knowledge to clinical education, which may serve as suitable media for medical learning. Finally, age, gender, and prior academic achievement might influence the correlations between each factor within MLSE scale, and our enrollees were predominantly male. Extrapolating our findings to other medical student populations needs careful adjudication.

## Data Availability

The raw data for conducting this analysis are unavailable due to administrative regulations.

## Abbreviations

AVE,: average variance extracted;
CFA,: confirmatory factor analysis;
CFI,: comparative fit index;
CR,: composite reliability;
CU,: conceptual understanding;
CVI,: content validity index;
EA,: everyday application;
EBM,: evidence-based medicine;
EFA,: exploratory factor analysis;
GFI,: goodness of fit index;
HC,: higher-order cognitive skill;
I-CVI,: item content validity index;
IFI,: incremental fit index;
MC,: medical communication;
ML,: Maximum Likelihood;
MLSE,: medical learning self-efficacy;
NFI,: normed fit index;
NTU-CM;: National Taiwan University College of Medicine;
PW: practical work;
RMSEA,: root mean square error of approximation;
S-CVI,: scale level-CVI;
SD,: standard deviation;
SGT,: small group tutorial;
SRMR,: standardized root mean square residual;
TFI,: Tucker-Lewis index
